# Causes of death and factors associated with early mortality of HIV-infected adults admitted to Korle-Bu Teaching Hospital

**DOI:** 10.11604/pamj.2017.27.48.8917

**Published:** 2017-05-18

**Authors:** Adriana Saavedra, Nia Campinha-Bacote, Maurice Hajjar, Ernest Kenu, Fizza Syeda Gillani, Adjoa Obo-Akwa, Margaret Lartey, Awewura Kwara

**Affiliations:** 1 Brown University, Providence, RI, USA; 2 Warren Alpert Medical School of Brown University, RI, USA; 3 Department of Medicine, Korle Bu Teaching Hospital, Korle-Bu Accra; 4 Department of Medicine, University of Ghana School of Medicine & Dentistry, College of Health Sciences, Accra, Ghana; 5 The Miriam Hospital, Providence, RI, USA; 6 University of Florida College of Medicine, Gainesville, FL, USA

**Keywords:** Cause of Death, HIV/AIDS, HAART, Ghana, Tuberculosis

## Abstract

**Introduction:**

This study sought to identify common causes of death as well as the factors associated with the high inpatient mortality rate of HIV-infected patients at the Korle-Bu Teaching Hospital (KBTH).

**Methods:**

The retrospective study reviewed the medical records of 547 HIV-infected adults aged 18 years or older admitted to the KBTH between the months of January 2012 and October 2013. Using standardized abstraction forms, clinical and demographic data of eligible patients was collected. Data was summarized using descriptive statistics. Demographic and clinical characteristics of patients who died within 7 days (early) and after (late) admission were compared using Rank Sum tests or Chi-square tests.

**Results:**

Of 547 eligible patients during the period, 222 (40.6%) died during hospitalization, with 124 (55.9%) of them dying within a week of admission. Of the 222 patients who died, 190 (85.6%) were previously known HIV-positive. Yet, 141 (63.5%) of the 222 patients who died had no prior highly active antiretroviral therapy (HAART). The most common admitting diagnoses were anemia (34.2%), cerebral toxoplasmosis (29.3%), and pneumonia (25.7%); the most common causes of death were tuberculosis (34.7%), anemia (30.2%) and cerebral toxoplasmosis (27.5%). Tuberculosis was the only factor significantly associated with early death (P<0.05).

**Conclusion:**

The inpatient mortality rate among HIV-infected adults admitted to the KBTH is high. A majority of the patients were not receiving HAART despite known HIV diagnosis. Earlier initiation of HAART may lower the risk of opportunistic infections and HIV mortality rates. Additionally, a high index of suspicion and initiation of empiric treatment for TB may reduce early deaths.

## Introduction

Human immunodeficiency virus (HIV) infection causes a large proportion of morbidity and mortality worldwide despite the availability of highly active antiretroviral therapy (HAART). Since the first outbreak of HIV in 1981, an estimated 39 million individuals have lost their lives to this illness [1]. However, there has been a decline as can be seen by the 35% drop in HIV-related deaths since its peak in 2005 [1]. Organizations such as UNAIDS and the World Health Organization (WHO) responded to the global burden of HIV/AIDS as they began to invest money and resources into combating this disease, focusing efforts on antiretroviral drug distribution. In 2013, US$19.1 billion was made available as a resource towards a global AIDS response, and in the same year an estimated 12.9 million people living with HIV/AIDS (PLWHA) had access to HAART [1]. Though significant progress has been made towards the goal of controlling HIV/AIDS worldwide, there still remains a disproportionate number of PLWHA, with 24.7 million (71%) of HIV cases residing in Sub-Saharan Africa [1]. Much of this disparity can be attributed to lack of resources and treatment available to populations of developing countries [2–5]. In 2013, in Western and Central Europe and North America, over half (51%) of PLWHA received treatment coverage in comparison to the 37% treatment coverage found in Sub-Saharan Africa [1]. Moreover, there is a stark difference in the common causes of mortality in HIV clinics between high-income and low- or middle-income countries [2, 4–9]. In a study published in 2015, researchers analyzed the 2011 medical admission records of the Oshakati Intermediate Hospital in Northern Namibia and discovered that 82% of patient deaths were a result of HIV/AIDS-related diseases [2].

Similarly, in other resourced-limited settings such as the Jos University Teaching Hospital in Nigeria and the Sassoon General Hospital in Pune India, a majority of deaths were caused by opportunistic infections, with tuberculosis being the most common [**3**]. In contrast, in settings within HIV clinics where HAART uptake is high, such as those in high-income countries like France and the United States, hospitals experience higher rates of HIV mortality due to non-infectious causes, with the most common being cardiovascular, pulmonary, and chronic liver disease [6, 8]. According to the WHO, a generalized epidemic is characterized by a disease prevalence of 1% or greater in the general population [10]. In Ghana, the median HIV prevalence for 2012 was 2.1% (CI 1.55-2.59), a rate over three times higher than the United States' median HIV prevalence of 0.65% [10, 11]. The Fevers Unit (HIV ward) of the Korle Bu Teaching Hospital (KBTH) is the largest national referral treatment center for individuals infected with HIV/AIDS in Ghana, receiving new cases as well as referrals of complicated cases from other HIV clinics for specialist care [12]. The mortality rate of the hospitalized patients at this center is high but to the best of our knowledge, the characteristics of the patients who die during hospitalization has not been previously investigated. The aim of this study was to investigate the most frequent admitting diagnosis and causes of death among the adults admitted to the KBTH Fevers Unit. In addition, we examined the demographic and clinical factors associated with inpatient deaths within one week of admission as such data may inform interventions to reduce mortality.

## Methods

**Study population and design:** This was a retrospective study that reviewed the medical records of HIV-infected adults aged older than 18 years who were hospitalized at the Fevers Unit of KBTH between January 2012 and October 2013. Demographics (age, gender, ethnicity, education level, current employment status), clinical data (presenting condition and symptoms, dates of presentation and discharge/death, HAART initiation, HAART regimens, opportunistic infections), and laboratory data (vitals, CD4 cell counts) were collected using standardized abstraction forms for HIV inpatients with known status and diagnosis upon admission. Regarding causes of death (COD), autopsy reports and medical charts were the primary sources, which were then compared against death certificates, when available. In cases of incomplete medical records, death was ascertained from the Fevers Unit register. Additionally, active tracking of patients having been admitted more than once to the clinic was undertaken and each admission was entered as a separate record in our data set. The two most widely used systems currently to classify cases of HIV is from the U.S. Centers for Disease Control and Prevention (CDC) classification system and the World Health Organization (WHO) Clinical Staging and Disease Classification System [13]. For the purposes of our study, we used the CDC disease staging system to categorize the severity of HIV disease [13]. According to the CDC, HIV-infected individuals are characterized as having acquired immunodeficiency syndrome (AIDS) with CD4 counts <200 cells/μL, and staging is determined by specific HIV-related conditions and symptoms [13]. Due to this staging, CD4 levels of patients and admitting conditions and symptoms were recorded, when available.

**Data management:** Data was entered into a Microsoft Access database containing electronic versions of the abstraction forms used. Data was then copied and imported into an excel workbook where it was cleaned and coded.

**Statistical analysis:** SigmaPlot™ version 12.5 was used to analyze the data. For numerical variables, age as well as median and interquartile ranges were calculated. Mann-Whittney Rank Sum test was used to compare continuous variables, and Chi-Squared test or Fischer Exact test was used to compare categorical variables between patients who died within 7 days and after 7 days of admission. For all analysis, P values < 0.05 were considered significant.

**Ethics:** This study was reviewed and approved by the Institutional Review Boards (IRB) for studies on human subjects of Brown University and the Ethical and Protocol Review Committee of the University of Ghana Medical School.

## Results

**Demographic characteristics:** Between January 2012 and October 2013, 547 HIV-infected patients were admitted to the KBTH Fevers Unit, of whom 222 (40.6%) died during hospitalization. Of the 222 patients who died, 170 (76.6%) patients were between the ages of 25 and 49, the mean age (SD) being 41.5 (9.8) years old; (Median 41.0, IQR 34.0-46.8) years old. Females made up 53.8% (120) of the study population; 103 (46.4%) were married or cohabitating and 111 (50.0%) were single, divorced, or separated (Table 1). Regarding known HIV status, 190 (85.2%) patients were previously known HIV-positive upon admission, yet only 70 (31.4%) were on HAART or had past exposure to HAART. Of the 125 patients with CD4 count data available, the median (IQR) CD4 count was 43 (15-114) cells/μL. Of the 222 patients who died, 124 (55.9%) died within a week (≤7 days) of admission.

**Table 1:** Baseline characteristics of the study population

Characteristics	Total (N=222)	N %
**Age Distribution**		
18-24	6	2.7
25-49	170	76.6
50 or older	44	19.8
Unknown	2	0.9
**Sex**		
Male	101	45.5
Female	120	54.1
Unknown	1	0.4
**Marital Status**		
Married/Cohabiting	103	46.4
Single/Divorced/ Separated	111	50.0
Unknown	8	3.6
**Education**		
Primary and JSS	53	23.9
Sec/Tech & MSLC	86	38.7
University	20	9.0
None	20	9.0
Unknown	43	19.4
**Past Exposure to HAART**		
Yes	70	31.4
No	141	63.2
Unknown	12	5.4
**HIV Status**		
Known Positive	190	85.6
Newly Diagnosed	31	14.0
Unknown	1	0.4
**Hospital Duration**		
≤ 7 days	124	55.9
> 7 days	96	43.2
Unknown	2	0.9

**Admitting diagnoses and causes of death:** Of the 222 patients who died, anemia was the most common diagnosis, with 76 (34.2%) patients being diagnosed with anemia upon admission. Cerebral toxoplasmosis (29.3%), pneumonia (25.7%), tuberculosis (20.3%), and HIV wasting syndrome (19.8%) were also among the top five admitting diagnoses (Table 2). Combined extrapulmonary and pulmonary TB was the leading cause of death with 77 (34.7%) patients dying from TB. Anemia (30.2%), cerebral toxoplasmosis (27.5%), pneumonia (23.0%), and gastroenteritis (10.4%) were also among the most common causes of death (Table 2).

**Table 2:** Common admitting diagnosis and causes of death

Admitting Diagnosis	Number (%)
Anemia	76 (34.2)
Cerebral Toxoplasmosis	65 (29.3)
Pneumonia	57 (25.7)
Tuberculosis *	45 (20.3)
HIV Wasting Syndrome	44 (19.8)
Gastroenteritis	28 (12.6)
TB Meningitis	4 (1.8)
**Causes of Death**	**Number (%)**
Tuberculosis	77 (34.7)
Anemia	67 (30.2)
Cerebral Toxoplasmosis	61 (27.5)
Pneumonia	51 (23.0)
Gastroenteritis	23 (10.4)

**Factors associated with early death in comparison to late death:** Due to the large proportion (55.1%) of inpatient deaths occurring within 7 days of admission, further analysis was conducted comparing factors associated with early death (≤7 day hospital duration) and late death (>7 day hospital duration). Tuberculosis was the only factor associated with early death (P = 0.04). All of the other factors evaluated were not significantly associated with early death (P>0.05) (Table 3).

**Table 3:** Analysis of demographic and clinical factors associated with early mortality during hospitalization at the Korle-Bu Teaching Hospital fevers unit

Characteristics	Early death (N =124 )	Late death (N = 96)	*P* value#
	**n (%)**	**n (%)**	
Median (IQR) age	41 (47)	42 (47)	0.811
Median (IQR) CD4 count	46(118.500)	40 (112.000)	0.547
Median (IQR) WBC	6.615(9.075)	5.000(8.300)	0.169
**Sex**			**0.523**
Female	64 (51.6)	55 (57.3)	
Male	59 (47.6)	41 (42.7)	
**HIV status**			**0.352**
Known	109 (87.9)	80 (83.3)	
New	14 (11.3)	16 (16.7)	
On HAART			0.468
Yes	43 (34.7)	27 (28.1)	
No	74 (59.7)	65 (67.7)	
**Cause of death**			
**Anemia**	**34 (27.4)**	**32 (33.3)**	**0.42**
**Cerebral Toxoplasmosis**	**30 (24.2)**	**29 (30.2)**	**0.40**
**Pneumonia**	**33 (26.6)**	**18 (18.8)**	**0.23**
**Gastroenteritis**	**13 (10.5)**	**10 (10.4)**	**0.837**
Tuberculosis^+^*	**33 (26.6)**	**39 (40.6)**	0.040*

+ Tuberculosis refers to both pulmonary and extrapulmonary cases

* Statistically significant at p<0.05

## Discussion

The main finding of this study is the high inpatient mortality rate among HIV-infected patients admitted to the HIV ward of the KBTH in the era of HAART with nearly half of the patients dying within 7 days of admission. In addition, TB was seen to be the most common cause of death as well as the only factor associated with early mortality. The high death rates predominantly due to treatable or preventable AIDS-associated conditions suggest that appropriate interventions focused on early HIV diagnosis with initiation of effective HAART could avert a majority of the deaths in this population. Anemia was the most frequent admitting diagnosis among the patients who died during admission at the KBTH. Several studies have shown significant associations between HIV infection and prevalence of anemia [14–18]. However, anemia is a multifactorial condition known to be caused by malaria, nutrient deficiencies, and hookworm infection, among other infections, making it difficult to isolate the specific role it plays in HIV infection [15]. Early antiretroviral therapy is expected to decrease the occurrence of anemia. Studies conducted at Jimma University Specialized Hospital and University of Gondar Hospital in Ethiopia revealed that overall HAART exposure decreased anemia prevalence in patients [15, 17]. Similarly, at an antiretroviral center in Cape Town, South Africa, HAART was shown to resolve 66% of anemia cases in HIV-positive patients after just one year of treatment [18]. As previously mentioned, anemia in HIV-infected patients, especially in low-resource settings, can be attributed to multiple causes, but it is likely an indicator of advanced HIV infection and delayed initiation of HAART in our settings. Our study was not designed to investigate the relationship between the prevalence of anemia and history of HAART, however, most of the patients in our study were not on HAART. While early HAART may reduce the frequency of hospitalization due to anemia or other HIV-associated conditions such as TB, efforts to reduce HIV morbidity and mortality should focus on early identification of HIV-infected patients and initiation of HAART. In a retrospective chart review of KBTH Fevers Unit inpatients in 2007, TB was found to be the most common cause of death, accounting for 57.7% of adult deaths [19]. In our cohort, we also found TB to be the most common cause of death (34.7%) as well as being the only factor significantly associated with early death (P<0.05). Numerous studies have investigated the interaction between HIV and TB infections [20–22], with one study stating, “HIV infection is the strongest risk factor for developing tuberculosis and has fuelled its resurgence, especially in sub-Saharan Africa” [23].

Similar to the studies focused on anemia, research has also shown significant association with HAART and reductions in TB incidence [23, 24]. The multinational INSIGHT START study and TEMPRANO study in Ivory Coast have shown that early HAART alone or early HAART with isoniazid preventive therapy (IPT) respectively reduced the risk of death or severe HIV-associated illness [25, 26]. Similarly, in another study conducted in Rio de Janeiro, researchers found that increasing TB screening and the implementation of IPT significantly reduced TB prevalence and mortality of HIV-infected patients [27]. Though much progress has been made with regards to TB screening, initial diagnosis, and treatment, it still lags behind the more rigorous HIV care and control programs [20]. Thus, early HIV diagnosis and treatment irrespective of CD4 count, early screening, and use of IPT in HIV-infected patients in Ghana may reduce overall AIDS-associated mortality. The overall mortality rate at the KBTH Fevers Unit was 31.5% as noted in published 2007 data [19]. Our current study found an even higher mortality rate of 40.6% among HIV-infected patients admitted to the unit. This rate is quite high especially given that with the advent and wide spread use of HAART, mortality rates have declined dramatically in high-income countries [28, 29]. The average life expectancy of a 20-year-old individual receiving HAART in a high-income country such as the U.S. or Canada is in the seventies, while in a low- or middle-income country like Namibia, life expectancy is capped at 44 years [2, 30]. Our findings suggest that earlier identification and treatment of individuals who require therapy through enhancement of HIV testing is essential in the reduction of HIV morbidity and mortality. In a study comparing 18 HAART programs in Africa, Asia, and South America with twelve HIV cohort studies in Europe and North America, results showed a mortality rate nearly three times greater for HIV-positive patients in low- or middle-income countries [31]. There is an undeniable disparity in the effective use of HAART regimens in high-income and low-income countries as the efficacy of these regimens are similar in both settings [32]. Early use of HAART is not only associated with better survival, but is also seen to reduce HIV transmission [33]. Thus, it is critical that steps are taken to implement more effective HIV screening and high quality HAART programs in low- and middle-income countries such as Ghana. Though our study did not examine the association between HAART and common conditions such as anemia or tuberculosis, our results show that delayed initiation of HAART in patients already known to have HIV could account for the high early inpatient death rates, illustrating the impact of early HAART in reducing mortality [25, 33]. It is essential to ensure that early HAART initiation happens in resource-limited settings. A study conducted in Côte d'Ivoire, cited by the WHO, found that HIV-positive individuals who took HAART immediately after diagnosis reduced the probability of contracting a severe illness by 44% when compared to those who took HAART only after their CD4 counts dropped below 500/mm^3^ [21].

The most recent published National AIDS Programme Guidelines for Antiretroviral Therapy in Ghana states that HIV-positive individuals can only start HAART treatment if they possess one or more of the following characteristics: their CD4 counts are less than 350 cells/ml; their symptoms can be categorized under WHO clinical stage 3 and 4; or if pregnant, they can be placed under HAART prophylaxis [22]. Moreover, patients must attend at least two pre-treatment counseling sessions with an HIV counselor to ensure that they understand the chronicity of HIV and the importance of HAART adherence [22]. In this study, a considerable number of medical records noted that patients did not complete pre-treatment counseling, impeding HAART initiation. This highlights the importance of a new paradigm where all HIV-infected patients are offered therapy as soon as possible and adherence sessions are tailored to patients' needs to ensure follow-up of newly diagnosed individuals rather than requiring mandatory pre-treatment counseling sessions. It is important to note that though the majority of the patients entering the KBTH Fevers Unit had medical records indicating prior HIV diagnosis, analysis of the exact date of diagnosis was not completed. It is very possible that due to KBTH's high intake of referral cases, many patients may have been recently diagnosed with HIV at the clinic they were referred from, not giving ample time to have met the aforementioned pre-treatment requirements. Thus, earlier HIV diagnosis through routine testing before patient becomes symptomatic should also be encouraged to help combat high mortality rates. It is also expected that identifying and combatting stigma may encourage earlier presentation as well as aid HAART initiation and adherence. A study conducted in Kumasi found that both PLWHA and caregivers were ostracized by family and community members because of their association with HIV [34]. Reasons for isolation stem from misinformation regarding HIV and the belief that severe reduction in social contact would prevent the spread of infection [34]. The organization of anti-stigma interventions and educational programs for family members can help communities become more knowledgeable with regards to HIV [34], ultimately helping to encourage earlier presentation and HAART initiation. Limitations of this study include difficulties in ascertaining information written within the medical records. Because this chart review was based primarily on physical medical records, there were times when handwritten admitting diagnoses and other information were illegible. In these cases, interpretation from KBTH physicians was solicited; however, this system was imperfect. Another limitation was high frequency of missing data in important CD4 counts, hemoglobin levels, and other laboratory tests. Due to the high frequency of early death (55.9%) died within a week of admission), the results of virological and immunological tests were often absent. Similarly, death certificates and autopsy results were rare, and causes of death were primarily extracted from handwritten post mortem diagnoses in the medical records.

## Conclusion

This study at the large inpatient unit of the KBTH demonstrates a high overall inpatient mortality rate of 41%. Anemia was identified as the most frequent admitting diagnosis and tuberculosis was identified as the most common cause of death, both conditions associated with delayed use of HAART in HIV-infected patients. Overall, the common causes of death are all treatable opportunistic or concurrent infections suggesting that mortality could be reduced by appropriate interventions. Measures should be taken to strengthen the early diagnosis and treatment of these common conditions by investing effort into the improvement of screening, initial diagnosis, and treatment. In the long term, early HIV diagnosis and initiation of HAART irrespective of CD4 count will reduce the frequency of the opportunistic infections, hospitalizations, and mortality. The high mortality rates in this cohort reflect the critical time between HIV diagnosis and HAART initiation among the infected persons in the community. Addressing stigma and educating communities about HIV is crucial to seeing reductions in HIV mortality, as early diagnosis, treatment, and presentation all continue to be essential methods in controlling HIV.

### What is known about this topic

- Where antiretroviral treatment uptake is high, non-infectious causes such as cardiovascular, pulmonary, and chronic liver disease are the most common causes of mortality among HIV-infected patients;
- A majority of HIV-associated deaths in resource-limited settings is due to AIDS-associated infections;
- A review of causes of death at the current study site in Ghana using 2007 data showed that opportunistic infections were the most common causes of death, with TB accounting for the majority of the deaths.

### What this study adds

- Despite scale up of antiretroviral therapy in Ghana in the last decade, cerebral toxoplasmosis, pneumonia and TB are the most common causes of HIV mortality;
- Tuberculosis was the only factor significantly associated with early death within a week of hospitalization;
- Our study suggests that not only is increased coverage of ART necessary in Ghana, early diagnosis of HIV and initiation of ART is critical to prevent opportunistic infections and death.

## Competing interests

The authors declare no competing interests.
